# Classification of colon adenocarcinoma based on immunological characterizations: Implications for prognosis and immunotherapy

**DOI:** 10.3389/fimmu.2022.934083

**Published:** 2022-07-27

**Authors:** Midie Xu, Jinjia Chang, Wenfeng Wang, Xin Wang, Xu Wang, Weiwei Weng, Cong Tan, Meng Zhang, Shujuan Ni, Lei Wang, Zhaohui Huang, Zhenzhong Deng, Wenhua Li, Dan Huang, Weiqi Sheng

**Affiliations:** ^1^ Department of Pathology, Fudan University Shanghai Cancer Center, Shanghai, China; ^2^ Department of Oncology, Shanghai Medical college, Fudan University, Shanghai, China; ^3^ Institute of Pathology, Fudan University, Shanghai, China; ^4^ Department of Medical Oncology, Fudan University Shanghai Cancer Center, Shanghai, China; ^5^ Shanghai Urological Cancer Institute, Cancer Institute, Fudan University Shanghai Cancer Center, Fudan University, Shanghai, China; ^6^ Wuxi Cancer Institute, Affiliated Hospital of Jiangnan University, Wuxi, China; ^7^ Department of Oncology, Xinhua Hospital, School of Medicine, Shanghai Jiaotong University, Shanghai, China

**Keywords:** colon adenocarcinoma, immune characteristics, prognosis, therapy response, immune subtype analysis

## Abstract

Accurate immune molecular typing is pivotal for screening out patients with colon adenocarcinoma (COAD) who may benefit from immunotherapy and whose tumor microenvironment (TME) was needed for reprogramming to beneficial immune-mediated responses. However, little is known about the immune characteristic of COAD. Here, by calculating the enrichment score of immune characteristics in three online COAD datasets (TCGA-COAD, GSE39582, and GSE17538), we identified 17 prognostic-related immune characteristics that overlapped in at least two datasets. We determined that COADs could be stratified into three immune subtypes (IS1–IS3), based on consensus clustering of these 17 immune characteristics. Each of the three ISs was associated with distinct clinicopathological characteristics, genetic aberrations, tumor-infiltrating immune cell composition, immunophenotyping (immune “hot” and immune “cold”), and cytokine profiles, as well as different clinical outcomes and immunotherapy/therapeutic response. Patients with the IS1 tumor had high immune infiltration but immunosuppressive phenotype, IS3 tumor is an immune “hot” phenotype, whereas those with the IS2 tumor had an immune “cold” phenotype. We further verified the distinct immune phenotype of IS1 and IS3 by an in-house COAD cohort. We propose that the immune subtyping can be utilized to identify COAD patients who will be affected by the tumor immune microenvironment. Furthermore, the ISs may provide a guide for personalized cancer immunotherapy and for tumor prognosis.

## Introduction

Colon adenocarcinoma (COAD) is a common malignant tumor (1, 2). According to the latest data released by the World Health Organization’s International Agency for Research on Cancer (IARC) in 2020, COAD was the third most commonly diagnosed cancer and the second leading cause of cancer death worldwide (3). Most patients with COADs are diagnosed with resectable tumors and are treated with excisional surgery plus adjuvant therapy, if necessary. For patients with advanced colorectal cancer, target therapy combined with chemotherapy (containing oxaliplatin or irinotecan) is the primary treatment strategy. However, the current first-line chemotherapy regimens often cause severe side effects, such as gastrointestinal reactions, immune system damage, and even bone marrow suppression (4). There is therefore an urgent need to develop effective treatment regimens with fewer side effects.

Due to the rapid advancements and the remarkable survival benefits in patients with a variety of tumors, tumor immunotherapy, including treatment with or the use of monoclonal antibodies, immune checkpoint inhibitors, cytokine therapy, tumor vaccines, and adoptive cell therapy, is now considered to be the fifth pillar of antitumor therapy after surgery, chemotherapy, radiation, and targeted therapy (5, 6). Based on the degree of immune infiltration of the tumor, tumors can be divided into categories of highly infiltrating “hot tumors”, “variable tumors” with rejection and immunosuppression, and non-infiltrating “cold tumors” (7). Manipulation of immune regulatory pathways has been demonstrated as effective in different subsets of tumors, especially in paradigmatic immune-sensitive/”hot” tumors, such as melanoma (8) and non-small cell lung cancer (9). This is because these tumors harbor high levels of tumor mutational burden (TMB) (10–13), CD8 lymphocyte infiltration (14, 15), and programmed death-ligand 1 (PD-L1) expression. Scientists have also tried various approaches to increase immune-mediated responses, such as messenger RNA (mRNA) vaccines to reprogram the tumor microenvironment (TME) and switch “cold” tumors to “hot” tumors (16). At present, immunotherapy has become a research hotspot in the field of COAD treatment. Immune checkpoint inhibitors (ICIs), such as nivolumab and pembrolizumab, were approved by the FDA for patients with unresectable high microsatellite instability (MSI-H) or deficient DNA mismatch repair (dMMR) COAD. Despite numerous attempts, immunotherapy for the treatment of COADs has presented challenges, however (17–19).

Immunotherapy for COAD is not as effective as for immune “hot” tumors because most COADs harbor a low tumor mutation burden and lack of immune cell infiltration. Approximately 80%–85% of COAD patients are considered “cold” tumors, with microsatellite stable (MSS) or low microsatellite instability (MSI-L) (called MSS/MSI-L colorectal cancer), indicating a lack of response to immune checkpoint inhibitors (ICIs) (18–20). COADs can be divided into hypermutated and non-hypermutated types at the genomic level (21). In general, the more mutations the tumor harbors, the higher the immunogenicity detected in the TME. Therefore, the non-hypermutated types harbor fewer immune cells in the TME and have lower immunotherapy efficacy (22). In addition, according to the consensus molecular subtype (CMS) system, only 14% of the COAD population is characterized by hypermutation, microsatellite instability (MSI), and highly activated immune system (23) and is therefore sensitive to ICIs. According to driver mutations, *TCGA* pan-cancer study stratifies COAD into four subtypes: chromosomal instability (CIN), genomically stable (GS), hypermutated-insertion deletion mutation (HM-indel), and hypermutated-single-nucleotide variant predominant (HM-SNV), which also defined cancers into six immune subtypes (C1–C6) (24). For effective treatment strategies, accurate immune molecular typing is needed to screen out patients with COAD who may benefit from immunotherapy and whose TME require reprogramming to increase immune-mediated responses.

In this study, we conducted a multi-cohort retrospective study and classified COAD into three distinct immune subtypes (ISs), based on consensus clustering of immune characteristics. We demonstrated the stability and reproducibility of this classification in three independents datasets. Each of the three ISs was associated with distinct molecular and cellular features, clinical outcomes, and therapeutic response. The identification of ISs may facilitate the optimal selection of COAD patients sensitive to immunotherapy.

## Materials and methods

### Patients and datasets

We collected the medical data of 1,267 patients with COAD from two online databases: *The Cancer Genome Atlas* (*TCGA*) database and Gene Expression Omnibus (GEO) database (including three datasets: GSE39582, GSE17538, and GSE72970). For *TCGA* cohort (*n* = 437), the RNA‐seq data, somatic mutation, and corresponding clinical information of cases with follow-up information were obtained using the GDC-client tool (https://portal.gdc.cancer.gov/). The microarray gene expression profiles and patients’ clinical data of datasets GSE39582 (*n* = 519), GSE17538 (*n* =187), and GSE72970 (*n* = 124) were downloaded from the GEO database (https://www.ncbi.nlm.nih.gov/geo/). Furthermore, we downloaded the gene expression profile of patients from the GSE72970 dataset, in which samples were obtained before treatment with different chemotherapy regimens (5-FU-based FOLFIRI/FOLFOX or anti-CLDN1 monoclonal antibody treatment), and calculated the therapeutic response of each IS. Gene IDs were converted into official gene symbols according to the Genome Reference Consortium Human Build 38 (GRCh38) assembly. Only genes with Transcripts Per Kilobase Million (TPM; calculated in relation to exon reads) greater than 0 in more than 50% of the samples were included for analysis. Patient informed consent existed in both the public databases, and this study was conducted in accordance with the Helsinki Declaration.

A series of tissue microarray (TMA) slides, which includes 223 patients with COAD that was stored in the tissue bank of Fudan University Shanghai Cancer Center (FUSCC), were used for immunohistochemistry (IHC) analysis. IHC analysis on these samples was approved by the Research Ethics Committee of FUSCC, and all patients provided informed consent.

## Discovery and validation of the COAD immune subtypes

**Figure 1:**
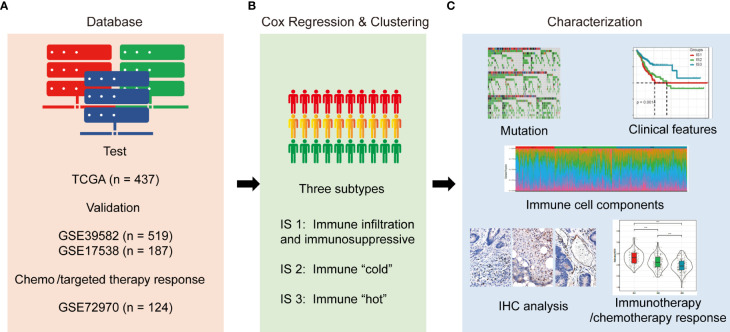
Study design and workflow of the present study. **(A)**. Four databases of COAD RNA-sequencing or gene microarray data were used as test or validation cohorts; **(B)**. RNA expression data were quantified with immune characteristics by univariate Cox regression analysis and hierarchically clustered into three subtypes; **(C)**. Mutation, clinical outcomes, immune characteristics, and enriched molecules were compared among the three subtypes. In addition, correlations between subtypes and responses to immunotherapy/chemotherapy were evaluated.

We calculated the enrichment score of immune characteristics in *TCGA*-COAD (**Supplementary Table 1**), GSE39582 (**Supplementary Table 2**), and GSE17538 (**Supplementary Table 3**) datasets using the IOBR TME-associated package in R software. The prognostic significance of the enrichment score was analyzed by performing univariate Cox regression analysis. Each of the immune characteristics related to disease-free survival (DFS) overlapped in at least two datasets we selected for further analysis (**Supplementary Tables 4** **-** **6**). We applied consensus clustering (25) to identify clusters of patients in robust immune subtypes (IS). Five hundred bootstraps with 80% item resampling were calculated based on the partition around medoids (PAM) classifier and Euclidean distance, the evaluated K-selected clustering was set between 2 and 10, and the optimal classification was determined by calculating a consistency matrix and a consistency cumulative distribution function. The ISs in the GSE39582 and GSE17538 datasets were then validated as follows: the in-group proportion (IGP) (26) and Pearson correlation among centroids of gene module scores were used to quantitatively measure the consistency and reproducibility of the acquired IS in the GSE39582 and GSE17538 cohorts. The study design and workflow are outlined in **Figure 1**. We analyzed the difference between the present ISs and other previous proposed COAD classification using a one-way ANOVA and the ssGSEA method.

## Evaluation of clinicopathological, molecular, and cellular characteristics associated with the IS

The disease-free survival (DFS) period of each COAD patient was calculated using the Kaplan–Meier method with the log-rank test and univariable Cox regression. Samples with survival time less than 30 days were excluded from the analysis. We downloaded the mutation data from *TCGA*-COAD dataset and calculated the TMB of each patient, then analyzed the distribution of TMB in each IS. Relationships between ISs and clinicopathological features, including age, sex, and histological type, were analyzed by non-parametric (Fisher’s exact) assessments, as appropriate.

## Evaluation of characteristics between molecular subtypes

We first calculated the gene expression of chemokines and chemokine receptors among the three ISs in *TCGA*-COAD cohort. Next, we obtained 47 immune checkpoint-related genes from the previous study (27) and analyzed their expression profiles among the three ISs. The innate immune cyclic GMP-AMP synthase (cGAS)/stimulator of interferon genes (STING) pathway has recently emerged as a nodal player in cancer immunity and is currently being explored as a potential therapeutic target (28). We compared the expression changes of four key genes in the innate immune cGAS/STING signal pathway, *CGAS* (encoding cGAS protein), *TMEM173* (encoding STING protein), and tank-bound kinase 1 (*TBK1*) and IFN regulator 3 (*IRF3*) (both are downstream effectors), among ISs using one-way ANOVAs and the ssGSEA method. We extracted Th1/IFN-γ gene signatures (27) and calculated the IFN-γ level of each patient among ISs by ssGSEA. Furthermore, we evaluated the intratumoral immune cytolytic activity (CYT) of each patient in *TCGA*-COAD cohort by calculating the average value of GZMA and PRF1 expression levels. Lastly, we obtained the angiogenesis-related gene set from the previous study (29) and evaluated the angiogenesis score of each patient.

In order to analyze the distribution of immune cell components in each IS, we determined the scores of 22 immune cells in each patient in *TCGA*-COAD, using the CIBERSORT database (30). In order to analyze the distribution of immune cell characteristics, especially T-cell components in each IS, we determined the enrichment score of 28 immune cells in each patient in *TCGA*-COAD cohort, by analyzing 28 immune cell marker genes using the ssGSEA method. The abundances of tumor-infiltration immune cells (B cells, plasma cells, T cells, NK cells, monocytes, mast cells, macrophages, eosinophils, neutrophils, and dendritic cell) were estimated from gene expression data, using the CIBERSORT database (https://cibersort.stanford.edu/) (30). The “Estimation of STromal and Immune cells in MAlignant Tumours using Expression data (ESTIMATE)” algorithm was applied to calculate the ImmuneScore and StromalScore, which represent the level of infiltrating immune cells and the presence of stromal cells in tumor tissues (31). The ESTIMATEScore is the sum of the ImmuneScore and StromalScore and refers to the purity of tumor tissues; the score specifies tumor cellularity in the TME. The T-cell dysfunction scores, T-cell rejection scores, and potential clinical efficacy of immune checkpoint inhibitors in each IS were evaluated using Tumor Immune Dysfunction and Exclusion (TIDE) software (http://tide.dfci.harvard.edu/) (32). Moreover, a high TIDE score is positively correlated with immune escape, and patients with a high TIDE score are less likely to benefit from ICIs (32). Therefore, we investigated the possibility of immune escape of each IS by calculating the TIDE score for TCGA-COAD cohort. Tumor-associated inflammation characteristics can promote tumor growth and progression by promoting angiogenesis and metastasis, subverting antitumor immune response, and changing the sensitivity of tumor cells to chemotherapeutic drugs (33–35). In order to analyze the distribution of inflammation characteristics in each IS, we determined the expression level of inflammation-related genes in each patient in *TCGA*-COAD cohort, using the ssGSEA method. We then analyzed the differences in the enrichment scores of the seven inflammation-related metagenes (*HCK*, *IgG*, *LCK*, *MHC-I*, *MHC-II*, *Interferon*, *STAT1*) among the ISs.

### Prediction of IS response to immunotherapy or chemotherapy

The R package “pRRophetic” was used to estimate the chemotherapeutic response of cisplatin and 5-fluorouracil in TCGA-COAD cohort (36). Gastrointestinal cancer cell lines and the “cgp2016” dataset were applied when implementing the “pRRopheticPredict” function. This methodology fitted the ridge regression model based on the drug sensitivity of the cell line and baseline of gene expression, thus predicting the chemotherapeutic response by using patients’ baseline gene expression data. Drug sensitivity was measured by the concentration required for 50% cellular growth inhibition (IC50). Based on genomic expression profiles and therapeutic and prognostic data in *TCGA-*COAD dataset, the potential response of each IS to traditional chemotherapy drugs cisplatin and 5-FU were predicted by the unsupervised subclass mapping (SubMap) method (37). In short, the IS of each sample was determined by analyzing their genomic expression profile, then the therapeutic and prognostic data of each sample were mapped (unsupervised subclass) to the IS, to predict the potential response of each IS to chemotherapy drugs.

## Analysis of the immune-related gene co-expression module

We clustered the 437 patients in *TCGA*-COAD cohort based on the expression of all genes with median absolute deviation (MAD) >50%, and the weighted gene correlation network analysis (WGCNA) co-expression algorithm was used to detect co-expressed gene modules using the R package WGCNA (38) (**Supplementary Figure S3A**). To ensure that the co-expression network can be a scale-free network, the co-expression modules were screened by setting a soft threshold power *β* as 10 (**Supplementary Figures S3B, C**). Among the gene co-expression modules obtained from cluster analysis and module fusion, gray modules represent gene sets that could not be merged. The topology overlap matrix (TOM) was then constructed from the adjacency matrix to avoid the influence of noise and spurious associations. Based on TOM, average-linkage hierarchical clustering using the dynamic shear tree method was conducted to define co-expression modules. The minimum gene size of each module was set to 60. To explore the relationship among modules, the feature vector values (eigengenes) of each module were calculated in turn, and modules with highly correlated eigengenes were merged into a new module through cluster analysis with the threshold as follows: height = 0.25, DeepSplit = 4, and minModuleSize = 60.

### Identification of hub genes by protein–protein interaction analysis

Since protein–protein interaction (PPI) analysis can help identify hub genes with core functions, PPIs among genes in the identified key modules were further explored. The Search Tool for the Retrieval of Interacting Genes (STRING) is a well-known database containing comprehensive PPI information (version 11.0, https://string-db.org/). The PPI network among these genes was thus mapped to the STRING assembly and then visualized by the Cytoscape software.

### Immunohistochemical staining

The expressions of angiogenesis marker-CD31, cytolytic activity marker-interferon-gamma (IFN-γ), and granzyme B (GZMB) of these CRC patients were also determined by IHC staining. IHC staining was performed as described previously (39). The primary antibodies are listed as follows: Anti-Interferon gamma antibody (Abcam, ab218426, 1:100), Granzyme B Monoclonal Antibody (Abcam, ab255598, 1:100), and CD31 (Gene Tech, M082304).

### Statistical analysis

All statistical analyses were performed using R 3.6.0 (https://mirrors.tuna.tsinghua.edu.cn/CRAN/) with default software parameters. A *P* value <0.05 was considered statistically significant. The biological function of genes in each immune gene co-expression module was annotated in Gene Ontology using the R package clusterProfiler. The Pearson correlation coefficient was used for correlation analysis. Univariate Cox regression analysis was performed to determine the immune-related gene co-expression modules with prognostic significance. A one-way ANOVA was applied for assessing the association between IS and the immune-related molecular and cellular characteristics using the ssGSEA method (24).

## Results

### Identification of potential immune subtypes of COAD

**Figure 2:**
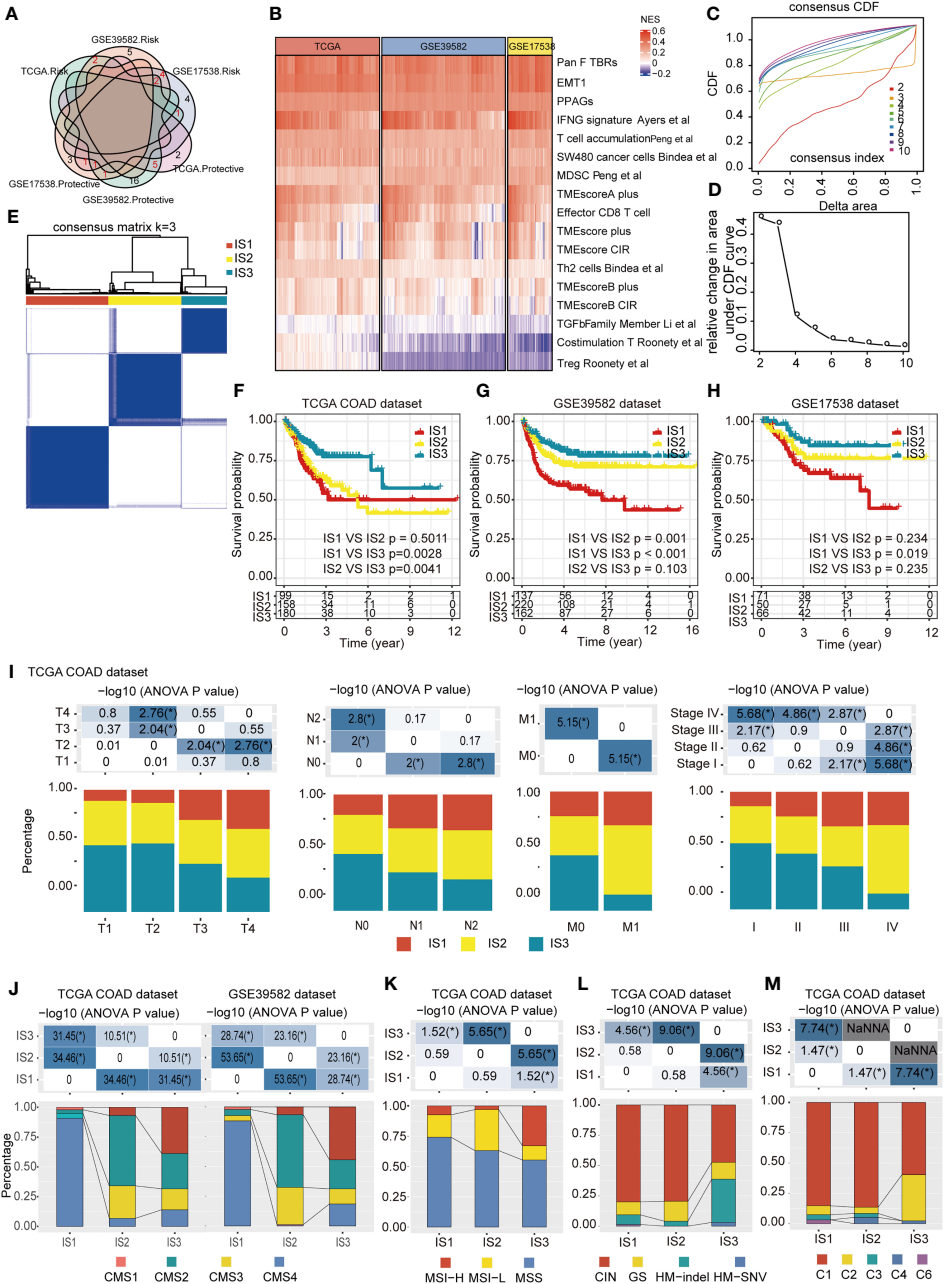
Identification of potential immune subtypes of COAD. **(A)**. Overlapping prognostic immune characteristics among *TCGA*-COAD, GSE39582, and GSE17538 cohorts; the lines correspond to different gene sets in each dataset; red numbers represent the intersection genes of different datasets. **(B)** The distribution of 17 immune characteristics among three cohorts; **(C, D)**. Cumulative distribution function (CDF) curve **(C)** and **(D)** delta area showed the stability of different cluster numbers in the consensus clustering result by using the enrichment score of the 17 immune characteristics. The consensus CDF diagram allows us to determine at what number of clusters, *k*, the CDF reaches an approximate maximum; thus, consensus and cluster confidence are at a maximum at this *k (Please See Xue.et al, PMID*: 19351533). In this manuscript, we set the *k* value = 3. **(E)**. Sample clustering heat map of the 437 samples in *TCGA*-COAD cohort. **(F–H)**. Kaplan–Meier curves with log-rank test showing DFS of ISs in *TCGA*-COAD **(F)**, GSE39582 **(G)** and GSE17538 **(H)** cohorts. **(I)**. Distribution of IS1-IS3 among the indicated clinicopathological characteristics in *TCGA*-COAD cohort. **(J)**. Distribution of IS1-IS3 among CMS classification in *TCGA*-COAD and GSE39582 cohort. **(K)**. Distribution of IS1–IS3 among patients with different microsatellite instability (MSI) statuses; IS2 and IS3 had the highest percent of patients with MSI-L and MSI-H subtypes, respectively. **(L)**. Distribution of IS1–IS3 among TCGA mutation classification; IS1 and IS2 are mainly composed of the CIN subtype, while IS3 showed more relevance with the HM-indel and HM-SNV subtype. **(M)**. Distribution of IS1–IS3 among TCGA immune subtypes; the IS1 and IS2 subtypes are mainly inclined to C1 subtypes, and the C6 subtype is mainly distributed within IS1, while the percent of C2 subtypes in IS3 was higher than that in IS1 and IS2. * P < 0.01, ** P < 0.001,*** P < 0.0001, and **** P < 0.00001.

We identified 14 disease-free survival (DFS)-related characteristics in *TCGA*-COAD cohort (**Supplementary Table 4**), 36 prognostic characteristics in the GSE39582 cohort (**Supplementary Table 5**), and 17 prognostic characteristics in the GSE17538 cohort (**Supplementary Table 6**), respectively. Specific immune characteristics varied among the three cohorts, with little overlapping characteristics (**Figure 2A**). From the DFS-related immune characteristics, 17 characteristics that were overlapped in at least two cohorts were included for subsequent analysis (*P* < 0.05, **Figure 2B**). By applying consensus clustering of 437 COAD samples using the enrichment score of these 17 DFS-related immune characteristics, we identified three molecular immune subtypes (ISs), IS1–IS3, in *TCGA-*COAD cohort (**Figures 2C–E**). Of these identified ISs, IS3 was associated with the longest DFS and IS1 with the shortest (**Figure 2F**). The ISs obtained from the datasets GSE39582 and GSE17538 displayed similar survival patterns (**Figures 2G, H**). There were significant differences in the distribution of patients’ clinicopathological characteristics, including T stage, N stage, M stage, and TNM stage among the three ISs in *TCGA*-COAD (**Figure 2I**) and GSE39582 cohorts (**Supplementary Figure S1B**), whereas there was no significant difference in the distribution of age and gender among the three ISs in both two datasets (**Supplementary Figures S1A, B**). In the GSE17538 cohort, there were significant differences in the distribution of patients’ TNM stage, histological grade, and gender among the three ISs (**Supplementary Figure S1C**). However, the same IS was differently distributed in these three cohorts, indicating the tumor heterogeneity. We further analyzed the distribution of four consensus molecular subtypes (CMS) (23) in these three ISs: IS1 consisted mostly of the CMS4 subtype, IS2 consisted mostly of the CMS2 subtype, and IS3 was more congruent with the CMS1 subtypes; CMS3 was mostly distributed in IS2 and IS3 (**Figure 2J**). When comparing the MMR status among each IS using TCGA COAD dataset, patients with the MSS status mostly fell into IS1, while IS2 and IS3 had the highest percentage of patients with MSI-L and MSI-H, respectively (**Figure 2K**). When analyzing the distribution of four TCGA pan-cancer mutation classification subtypes (CIN, GS, HM-indel, and HM-SNV) in these three ISs, IS1 and IS2 consisted mostly of the CIN subtype, while IS3 was more congruent with the HM-indel and HM-SNV subtypes (**Figure 2L**). We further compared the results of ISs with the six previous immune subtypes (C1~C6), which was also defined by *TCGA* pan-cancer study, and discovered that the identified IS1 and IS2 subtypes are most similar to C1 subtypes, whereas the C6 subtype is mainly distributed within IS1. Moreover, in comparison to IS1 and IS2, the percent of C2 subtypes was highest in IS3 (**Figure 2M**).

## The relationship between IS, tumor mutation burden, and common gene mutations in TCGA-COAD dataset

The TMB was significantly higher in IS3 than in IS1 or IS2, whereas no significant difference was observed between IS1 and IS2 (**Supplementary Figure S2A**). Additionally, there were 12,744 genes with mutation frequency >3 in at least one of all three ISs (**Supplementary Table 7**), and 5,414 genes showed a significantly different mutation frequency among the three ISs (*P* < 0.05, chi-square test; **Supplementary Table 8**). The number of gene mutations in IS1 and IS2 subtypes was significantly lower than that of IS3, whereas no significant difference was observed between IS1 and IS2 (**Supplementary Figure S2B**). Additionally, among the 10 mutation characteristics with the highest mutation frequency in each subtype, the proportion of adenomatous polyposis coli (*APC*) mutations in IS2 was significantly greater than for IS1 and IS3; the proportion of *TP53* mutations in IS1 was significantly higher than for IS2 and IS3; while the proportion of *KRAS* mutations in IS1 was significantly lower than in IS2 and IS3 (**Supplementary Figure S2C**). The frequency of the DNA polymerase ε (POLE) mutation showed no significant difference between the IS1 and IS2 subtypes but was significantly higher in the IS3 subtype (**Supplementary Table 8**).

## Distribution of immune-related molecular characteristics among IS by using TCGA-COAD dataset

**Figure 3:**
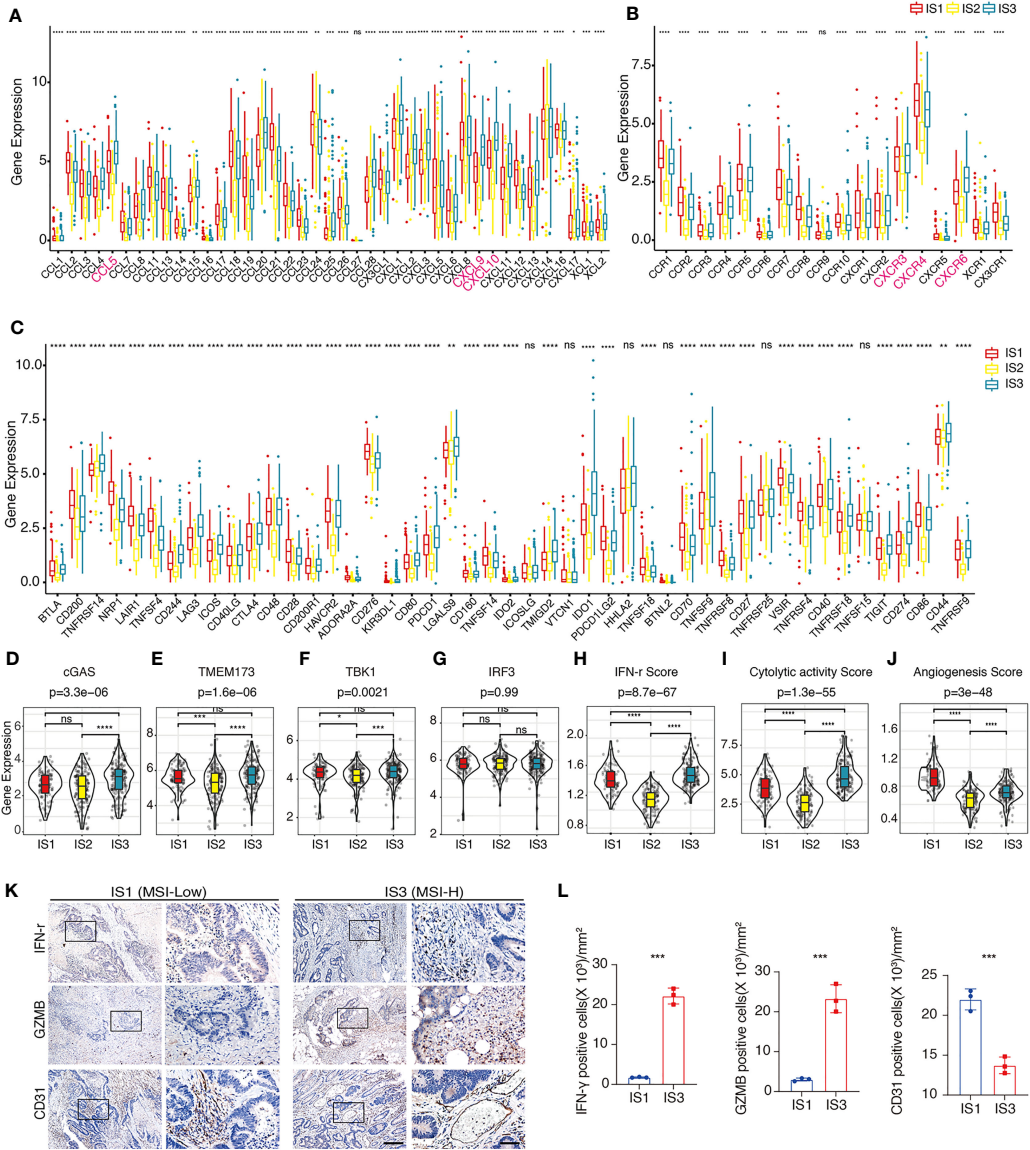
Distribution of immune-related molecular characteristics among ISs in TCGA-COAD and FUSCC cohorts **(A, B)**. Differential expression of chemokines **(A)** or chemokine receptors **(B)** among the COAD immune subtypes in TCGA-COAD cohort. *CCL4*, *CCL5*, *CXCL9*, *CXCL10*, *CXCR3*, *CXCR4*, and *CXCR6* are highlighted in pink. The top and bottom of the box are the upper quartile (Q3) and the lower quartile (Q1) of the data, respectively. The solid line in the box represents the median. The whiskers represent the maximum and minimum values of this group of data. The Kruskal–Wallis test was used to assess for significant differences. ns not significant, *P < 0.01, **P < 0.001, ***P < 0.0001, and ****P < 0.00001. **(C)**. Differential expression of immune checkpoint-related genes among the COAD immune subtypes in TCGA-COAD cohort. The top and bottom of the box are the upper quartile (Q3) and the lower quartile (Q1) of the data, respectively. The solid line in the box represents the median. The whiskers represent the maximum and minimum values of this group of data. The Kruskal–Wallis test was used to assess for significant differences. ns not significant, *P < 0.01, **P < 0.001, ***P < 0.0001, and ****P < 0.00001. **(D–G)**. Differential expression of *cGAS* **(D)**, *TMEM173* **(E)**, *TBK1* **(F)**, and *IRF3* **(G)** among the COAD immune subtypes in TCGA-COAD cohort. We used the Kruskal–Wallis test and Wilcox test to compare the significance among the three groups and pairwise comparison between groups, respectively. The solid black line in the box represents the median, and the black box in the violin plot represents the quartile range. The black vertical line running through the violin chart represents the interval from the minimum value to the maximum value, respectively. ns not significant, *P < 0.01, **P < 0.001, ***P < 0.0001, and ****P < 0.00001. **(H–J)**. The estimated IFN-γ level **(H)**, CYT level **(I)**, and angiogenesis level **(J)** among the COAD immune subtypes in TCGA-COAD cohort. We used Kruskal–Wallis test and Wilcoxon test to compare the significance among the three groups and pairwise comparison between groups, respectively. The solid black line in the box represents the median, and the inner black box in the violin plot represents the quartile range. The black vertical line running through the violin chart represents the interval from the minimum value to the maximum value, respectively. ns not significant, *P < 0.01, **P < 0.001, ***P < 0.0001, and ****P < 0.00001. **(K)**. Representative IHC result of IFN-γ, GZMB, and CD31 in dMMR and pMMR subtypes in the FUSCC cohort. The box area is magnified in the right panel. Scale bars: 100 µm (left panel) and 20 µm (right panel). **(L)**. Scatter plots show the difference of IFN-γ, GZMB, and CD31 in dMMR and pMMR subtypes in the FUSCC cohort. Unpaired t-test. Data are shown as mean ± SD. ***P < 0.0001.

The gene expression of chemokines and chemokine receptors among these three ISs showed that the expression of most (30/41) chemokines, such as CCL4, CCL5, CXCL9, and CXCL10, in IS2 was significantly lowest among all three IS (**Figure 3A**). *CXCR6* and the other most chemokine receptors (17/18) were significantly lower in IS2 compared to the other two ISs (**Figure 3B**). The expression of 41 (87.2%) of the immune checkpoint-related genes was significantly lower in IS2 than in IS1 and IS3, including *LAG3, ICOS, CTLA-4, HAVCR2 (TIM3), PDCD1*, and *CD274* (*PD-L1*) (**Figure 3C**). For the four genes in the innate immune cGAS/STING signal pathway, CGAS was significantly higher in IS3 than in IS1 and IS2, whereas the expression of TMEM173 and TBK1 was significantly lower in IS2 than in IS1 and IS3, and there was no significant difference in IRF3 expression level among the three ISs (**Figures 3D**–**G**). The expression level of IFN-γ was lowest in IS2, while it was the highest in IS3 (**Figure 3H**). IS2 had the lowest intra-tumoral immune cytolytic activity (CYT) level, and IS3 the highest (**Figure 3I**). The angiogenesis level of IS1 was significantly higher than that of IS2 and IS3 (**Figure 3J**). The data from the pathological archive showed that in the 223 patients with CRC we enrolled for IHC, 12 cases were dMMR (MSI-H) and the other 211 cases were pMMR (MSS) (**Table 1**). The expressions of CD31, IFN-γ, and GZMB in 12 randomly selected pMMR cases were compared with these 12 dMMR samples by IHC, which indicated that IFN-γ and GZMB were expressed robustly in patients with a dMMR status, while the angiogenesis marker CD31 expressed more strongly in patients with pMMR status (**Figures 3K**, **L**).

**Table 1:** Clinicopathological features of colorectal cancer patients.

Characteristics		n	%
Age (years)		233	
	<60	106	45.49%
	≥60	117	50.21%
Gender			
	Male	129	55.36%
	Female	94	40.34%
Primary cancer site			
	Colon,	124	53.22%
	Rectum	96	41.20%
	Unspecified	3	1.29%
Tumor size			
	<5cm	138	59.23%
	≥5cm	85	36.48%
Tumor differentiation			
	Well	1	0.43%
	Moderate	165	70.82%
	Poor	50	21.46%
	Unspecified	7	3.00%
Vascular invasion			
	Positive	38	16.31%
	Negative	185	79.40%
Perineural invasion			
	Positive	48	20.60%
	Negative	175	75.11%
Staging at diagnosis			
	Stage II	193	82.83%
	Stage III	30	12.88%
Primary tumor size			
	pT2	3	1.29%
	pT3	80	34.33%
	pT4	140	60.09%
Involvement of lymph node			
	pN0	193	82.83%
	pN1	20	8.58%
	pN2	10	4.29%
MMR status			
	dMMR	12	5.15%
	pMMR	211	90.56%

## Immune characteristics of ISs by using TCGA-COAD dataset

**Figure 4:**
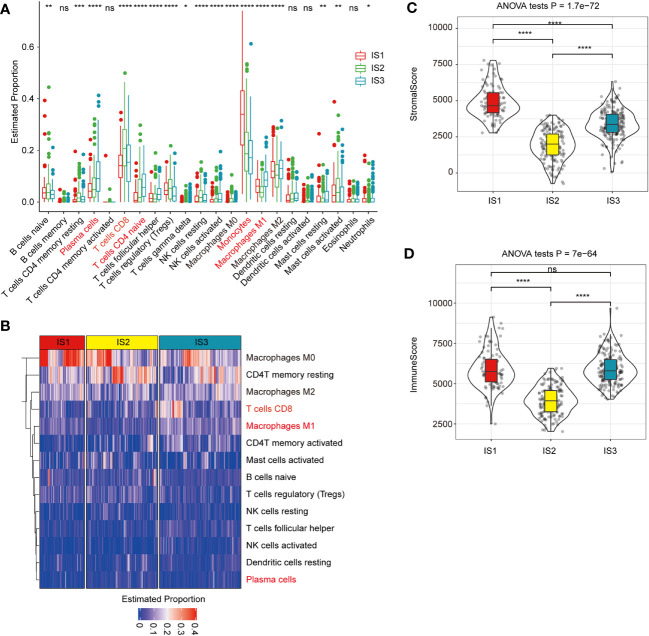
Association between immune subtypes and COAD-related tumor biomarkers in TCGA-COAD dataset **(A)**. The estimated proportion of immune cell infiltration among immune subtypes. CD8 T cell is highlighted in pink. The top and bottom of the box are the upper quartile (Q3) and the lower quartile (Q1) of the data, respectively. The solid black line in the box represents the median. The whiskers represent the maximum and minimum values of this group of data. The Kruskal–Wallis test was used to assess for significant differences. ns, not significant, *P < 0.01, **P < 0.001, ***P < 0.0001, and ****P < 0.00001. **(B)** Heat map for the estimated proportions of immune cells in the samples among immune subtypes. CD8+ T cell is highlighted in pink and significantly higher in IS3 subtypes. **(C, D)**. The proportions of StromalScore **(C)** or ImmuneScore **(D)** among immune subtypes in *TCGA*-COAD cohorts. IS1 has the highest relative proportion of stromal cells in TME, while IS2 has the lowest relative proportion of immune cells. We used the Kruskal–Wallis test and Wilcoxon test to compare the significance among the three groups and pairwise comparison between groups, respectively. The solid black line in the box represents the median, and the inner black box in the violin plot represents the quartile range. The black vertical line running through the violin chart represents the interval from the minimum value to the maximum value, respectively. ns, not significant, *P < 0.01, **P < 0.001, ***P < 0.0001, and ****P <0.00001.

The distribution of most immune cell components differed among the three ISs (**Figures 4A**, **B**). For example, monocytes in IS1 were significantly higher than those in IS2 and IS3, CD8^+^ T cells in IS1 were significantly lower than in IS2 and IS3, while CD4^+^ naïve T cells, plasma cells, and macrophages M1 in IS3 were significantly higher than in IS1 and IS2 (**Figures 4A**, **B**). The relative proportion of stromal cells in all ISs showed that IS1 had the highest relative proportion of stromal cells in TME, while IS2 had the lowest relative proportion of immune cells (**Figures 4C**, **D**).

## Distribution of immune cell and inflammation characteristics among ISs

**Figure 5:**
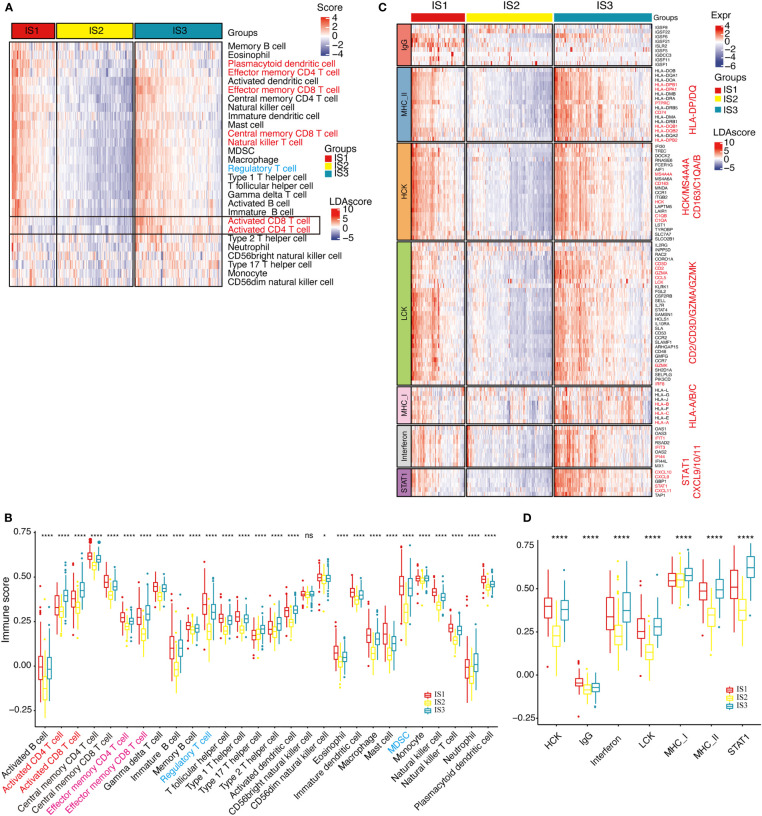
Distribution of immune cell characteristics and inflammation characteristics among ISs. **(A, B)**. Heat map **(A)** or boxplot **(B)** show the differential enrichment scores of 28 immune cell signatures among immune subtypes in *TCGA*-COAD cohorts. Activated CD4T, CD8T cells (highlighted with orange) are predominantly infiltrated in the IS3 subtype, while regulated T cells and MDSC cells (both are immunosuppressive cells, highlighted in blue) are predominantly infiltrated in the IS1 subtype. The top and bottom of the box are the upper quartile (Q3) and the lower quartile (Q1) of the data, respectively. The solid line in the box represents the median. The whiskers represent the maximum and minimum values of this group of data. The Kruskal–Wallis test was used to assess for significant differences. n.s, not significant, *P < 0.01, **P < 0.001, ***P < 0.0001, and ****P < 0.00001. **(C)**. Heat map for the expression level of inflammation-related genes in each patient among immune subtypes in *TCGA*-COAD cohorts. Marker genes are highlighted in orange. **(D)**. Differential enrichment scores of all seven inflammation-related metagenes among immune subtypes in *TCGA*-COAD cohorts. The IS3 subtype has the highest enrichment scores with *LCK, MHC-I, MHC-II*, and *STAT1* gene clusters. The top and bottom of the box are the upper quartile (Q3) and the lower quartile (Q1) of the data, respectively. The solid line in the box represents the median. The whiskers represent the maximum and minimum values of this group of data. The Kruskal–Wallis test was used to assess for significant differences. ns, not significant, *P < 0.01 and ****P < 0.00001.

In *TCGA*-COAD cohort, the enrichment scores of most immune cell components in IS1 and IS3 were significantly higher than in IS2, such as activated CD8^+^ and CD4^+^ T cells, effector memory CD8^+^ and CD4^+^ T cells, macrophages, and MDSCs (**Figures 5A**, **B**). Overall, the enrichment scores of most of immune cells in IS1 and IS3 were significantly higher than in IS2. The expression level of inflammation-related genes, which can be categorized into seven inflammation-related metagenes (MHC-I, MHC-II, HCK, LCK, interferon, STAT1, and IgG), showed that the majority of genes in MHC-II (HLA-DP, HLA-DQ, CD74), HCK (HCK, MA4A4A, CD163, C1QA, C1QB), LCK (LCK, CD2, CD3, GZMA, GZMK), MHC-I (HLA-A/B/C), interferon (IFIT1, IFIT3, IFI44), and STAT1 metagenes were highly expressed in IS1 (**Figure 5C**). With the exception of IgG, the enrichment scores of all the other six metagenes in IS1 and IS3 were significantly higher than that in IS2 (**Figure 5D**).

## The predicted IS response to immunotherapy and chemotherapy

**Figure 6:**
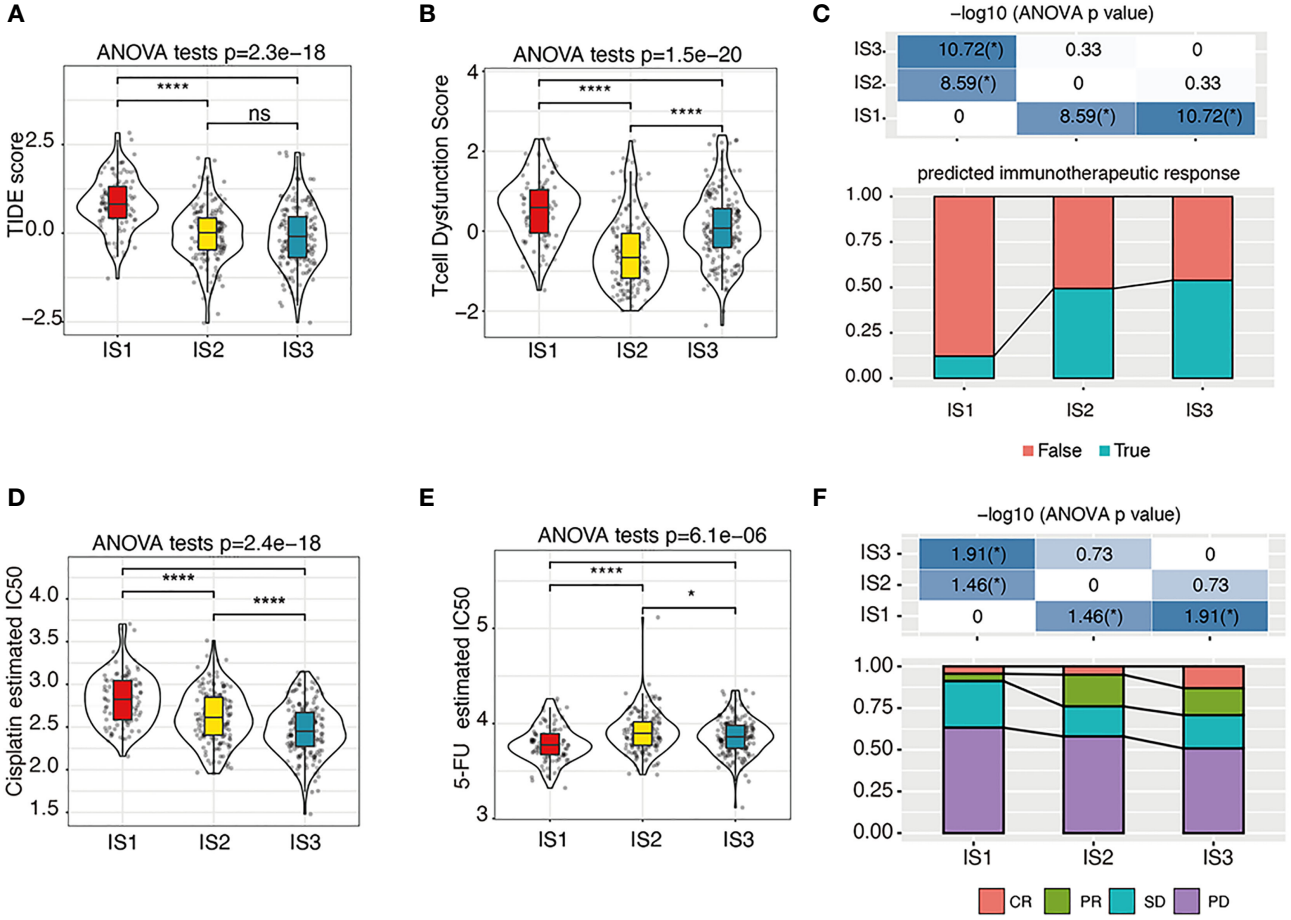
The immunotherapy/chemotherapy response of each IS. **(A–C)**. The estimated TIDE score **(A)**, T-cell dysfunction scores **(B)**, and predicted immunotherapeutic response statues **(C)** among immune subtypes. We used the Kruskal–Wallis test and Wilcoxon test to compare the significance among the three groups and pairwise comparison between groups, respectively. The solid black line in the box is the median, and the inner black box in the violin plot represents the quartile range. The black vertical line running through the violin chart represents the interval from the minimum value to the maximum value, respectively. n.s, not significant, *P < 0.01 and ****P < 0.00001. **(D, E)**. The predicted cisplatin **(D)** and 5-FU **(E)** chemotherapeutic response statues among immune subtypes. We used the Kruskal–Wallis test and Wilcoxon test to compare the significance among the three groups and pairwise comparison between groups, respectively. The solid black line in the box is the median, and the inner black box in the violin plot represents the quartile range. The black vertical line running through the violin chart represents the interval from the minimum value to the maximum value, respectively. n.s, not significant, *P < 0.01 and ****P < 0.00001. **(F)**. The response statues among immune subtypes in the GSE72970 cohort. PR, partial response; CR, complete response; SD, stable disease; PD, progressive disease. *P < 0.01.

The TIDE score was calculated to predict the possibility of immune escape of each IS using TCGA-COAD cohort, which showed that IS1 had the highest TIDE score (**Figure 6A**) and the highest predicted T-cell dysfunction score (**Figure 6B**). In addition, the proportion of predicted immunotherapy responses in IS1 was significantly lower than that of IS2 and IS3 (**Figure 6C**). These data suggest that the IS1 subtype is less likely to benefit from the anti-PD-L1 therapy. Besides, by analyzing the gene expression profile of TCGA-COAD cohort, the IS3 subtypes are predicted to be more sensitive to cisplatin than other ISs (**Figure 6D**), while IS1 is more sensitive to 5-FU (**Figure 6E**). Moreover, the gene expression profile and therapeutic response of patients from the GSE72970 dataset showed that, in patients who underwent 5-FU-based chemotherapy, the ratio of partial response (PR) cases in IS2 and IS3 was significantly higher than that in IS1, and the ratio of complete response (CR) cases in IS3 was significantly higher than that in IS1 and IS2 (**Figure 6F**).

## Function and prognosis analysis of co-expression gene modules among ISs

**Figure 7:**
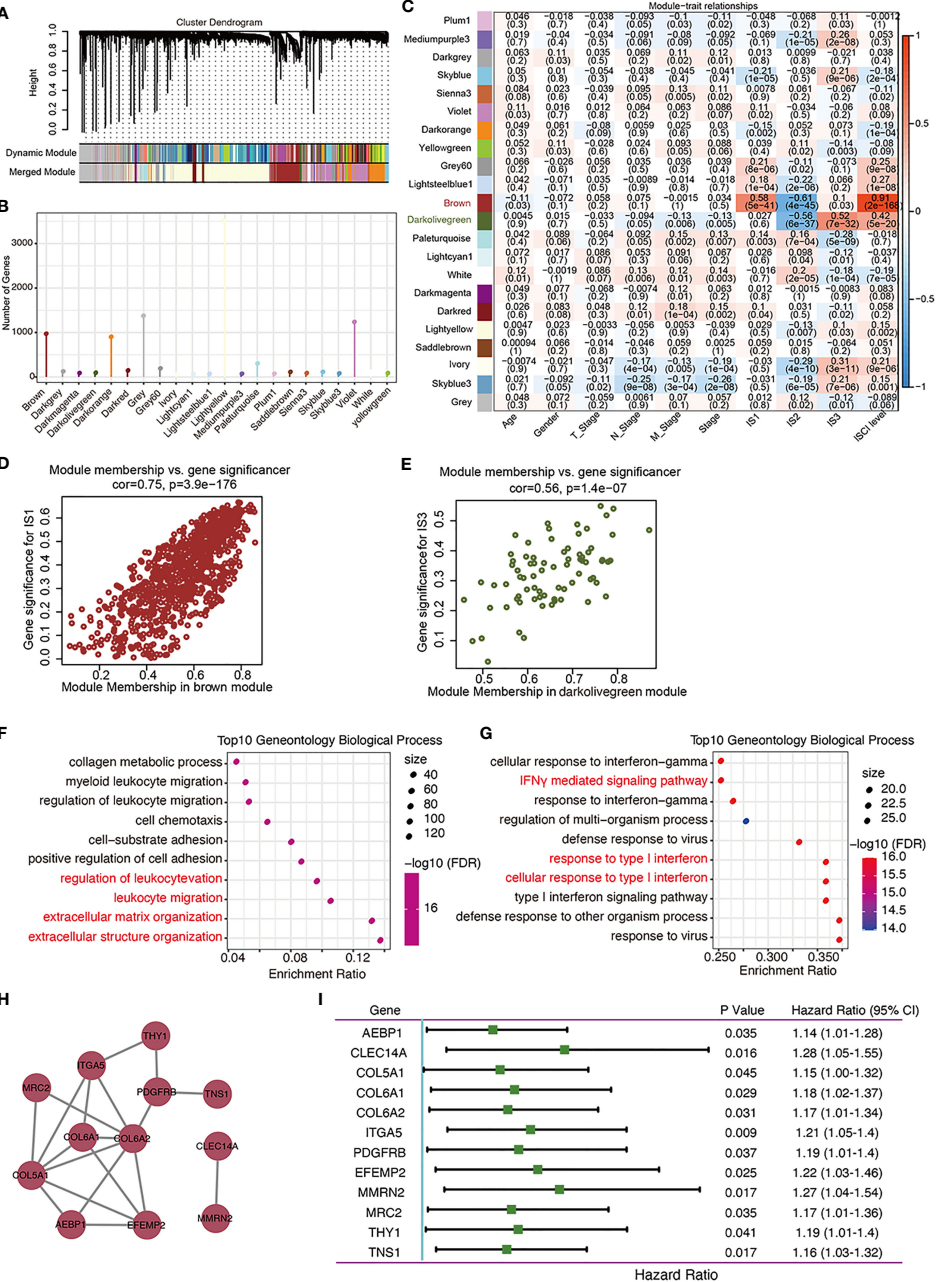
Identification of immune gene co-expression modules of COAD. **(A)** Dendrogram of all differentially expressed genes clustered based on a dissimilarity measure. (**B**). Gene numbers in each module. **(C–E)**. Evaluation of the distribution of these 22 modules in each clinicopathological feature and immune subtype **(C)**. The brown module was positively correlated with IS1 **(D)** and negatively correlated with IS2, while the darkolivegreen module was negatively correlated with IS2 and positively correlated with IS3 **(E)**.**(F, G).** Dot plot showing top 10 gene ontology biological processes in the brown **(F)** and darkolivegreen **(G)** module. **(H)**. Protein–protein interaction network of 12 DFS-related hub genes: *AEBP1, CLEC14A, COL5A1, COL6A2, ITGA4, PDGFRB, EFEMP2, MMRN2, MRC2, THY1*, and *TNS1*. **(I)**. Forest plot of the univariate Cox regression analyses for the prognosis value of the indicated 12 genes in *TCGA-*COAD cohort.

By clustering the 437 cases in *TCGA*-COAD cohort, a total of 22 gene co-expression modules were obtained after cluster analysis and module fusion (**Figure 7A**; **Supplementary Figures S3A–C**, **Supplementary Table 9**). Gene numbers in each module are shown in **Figure 7B**. The distribution of these 22 modules in each clinicopathological feature and IS was further evaluated, which showed that the brown module was positively correlated with IS1 (**Figures 7C, D**), while it was negatively correlated with IS2 (**Figure 7C**); the darkolivegreen module was negatively correlated with IS2 (**Figure 7C**) and positively correlated with IS3 (**Figures 7C, E**). Functional enrichment analysis showed that the brown module was related to leukocyte activation regulation, leukocyte migration, and extracellular matrix or structure organization (**Figure 7F**). The darkolivegreen module was related to immune-related pathways, such as the cellular response to IFN-γ, response to type I interferon (IFN), and IFN signaling pathway (**Figure 7G**).

Correlation analysis showed that 25 genes from the brown module (**Supplementary Table 10**) while no gene from the darkolivegreen module (**Supplementary Table 11**) were significantly correlated with brown module (r > 0.85) and DFS (*P* < 0.05). From these 25 genes, 12 hub genes (*AEBP1, CLEC14A, COL5A1, COL6A2, ITGA4, PDGFRB, EFEMP2, MMRN2, MRC2, THY1*, and *TNS1*) were obtained by constructing a PPI network, and the other genes with no interaction were excluded (**Figure 7H**). Univariate and multivariate Cox regression analyses results showed that the collated hub genes can predict the DFS of patients in *TCGA-*COAD cohort (**Figure 7I**). Thus, these 12 genes were selected as the final module feature gene. These hub genes can act as biomarkers for screening of the high-risk COAD population in IS1 and IS2.

## Discussion

COAD remains one of the most common malignancies worldwide, whereas the efficacy of current systemic treatment options is still limited (2, 4). At the time of admission, approximately 20%–25% of COAD patients are diagnosed with metastatic disease (40), and 25% develop locally recurrent or metastatic disease within 5 years. The 5-year survival of patients with metastatic COAD is only 15% (41). It is thus critical to investigate novel therapeutic targets so as to apply new treatments with improved clinical efficacy, whereas new immunotherapy techniques are not effective for all cancer patients. Accurate immune molecular typing is pivotal for screening out patients with COAD who may benefit from immunotherapy and whose TME requires reprogramming to increase immune-mediated responses. In the current study, we presented a comprehensive characterization of the immunological profile of COADs. Using *TCGA*-COAD dataset, we found that COADs can be stratified into three ISs, based on consensus clustering of immune characteristics. This IS stratification was confirmed using the GSE39582 and GSE17538 datasets as validation cohorts. These results indicate that the three molecular subtypes, based on immune characteristic enrichment scores, were reproducible in different COAD cohorts. Each of the three ISs was associated with distinct clinicopathological characteristics, genetic aberrations, tumor-infiltrating immune cell composition, immunophenotyping (immune “hot” and immune “cold”) (42), cytokine profiles, and different clinical outcomes and immunotherapy/therapeutic response. Our study suggests that identification of IS may facilitate the optimal selection of COAD patients responsive to adequate therapeutic strategies.

The innate immune cyclic GMP-AMP synthase (cGAS)/stimulator of interferon genes (STING) pathway has recently emerged as a nodal player in cancer immunity and is currently being explored as a potential therapeutic target (28). Our data showed that CGAS was significantly highest in IS3, suggesting that the cGAS/STING signal pathway is more active in IS3. IS3 also had the highest IFN-γ level, which can be produced by CD8^+^ T cells and inducing the overexpression of the *PD-L1/PD-L2* gene (43, 44). In addition, IS3 showed significantly higher CD4^+^ T-cell, CD8^+^ T-cell, and macrophage M1 percentages among the three ISs. These findings suggest that the immune characteristics displayed by IS3 present a classic immune “hot” phenotype, which is sensitive to immunotherapy (42), and the variation in COAD prognosis may be related to the distribution of these cell types. The tumor microenvironment (TME) of IS1 showed composite immune signatures reflecting a high immune cell component, including macrophages, activated B cells, activated CD8^+^ T cells, effector memory CD8^+^ T cells, immature B cells, and MDSCs, indicating that IS1 acts as a high immune infiltration (immune “hot”) (7) phenotype. However, IS1 conferred the poorest DFS. Stromal cells have been reported as key contributors to an immunosuppressive TME and hinder antitumor immunity (45, 46). IS1 had the highest angiogenesis level and proportion of stromal cells, which both suggests a more invasive and metastatic potential of tumors (47) and is therefore linked with a poor prognosis. It has been reported that the tumor immune dysfunction and exclusion (TIDE) score positively predicts the possibility of immune escape, with a high TIDE score positively correlated with immune escape and a lower chance of patients benefiting from ICIs (32). IS1 had the highest TIDE score and predicted T-cell dysfunction scores in TME, as well as the highest tumor immune dysfunction and exclusion score, which was more similar to immune escape. Thus, IS1 may also be an “immunosuppressive” phenotype. In comparison, the majority of cases in IS2 were MSS/MSI-L, which showed the lowest relative proportion of immune cells, which was potentially due to the low checkpoint-related gene expression levels. It has been reported that a high CCL4/CCL5/CXCL9/CXCL10 expression is strongly associated with CD8^+^ T-cell infiltration and T-cell activation (48–51). Moreover, CXCL9, CXCL10, and CXCL11/CXCR3 axis play a central role in immune activation (52, 53). *CXCR6*, which was reported to be exclusively expressed on intratumoral CD8^+^ T cells in colon cancer, positions cytotoxic T cells to receive critical survival signals in the tumor microenvironment (54, 55). Our data showed that the expression levels of all these genes were significantly lower in the IS2 subtype among the three ISs, which suggest that IS2 has the lowest minimal immune activation; it tends to be an immune “cold” phenotype. In patients within this subtype, a combination of therapies aimed at converting the “cold” tumor to a “hot” tumor, with another immunotherapy or chemotherapy, might modulate both the host immune response and the TME toward a state more conducive to successful therapy. Moreover, in addition to harboring the highest adenomatous polyposis coli (*APC*) mutations and low *KRAS* and *TP53* mutations, IS2 have the most gene mutations. This suggests that, although the IS2 tumor may derive primarily from APC mutation, the pathogenesis of tumors in this subtype may be much more complex than the other ISs. Further functional and mechanistic studies of the mutated genes may identify the pathogenesis and therapeutic targets.

We further analyzed the distribution of four consensus molecular subtypes (CMS) (23) in these three ISs: IS1 consisted mostly of the CMS4 subtype, IS2 consisted mostly of the CMS2 subtype, and IS3 was more congruent with the CMS1 subtypes; CMS3 was mostly distributed in IS2 and IS3. The CMS4 subtype was characterized by a high stromal infiltration, TGF-β activation, and angiogenesis (23), and all these are also prominent features of IS1. Similarly, the IS3 subtype with better prognosis has a larger proportion of CMS1, both subtypes are characterized by high immune infiltration, and MSI-H CRC is mainly a feature in CMS1 (23) and IS3 subtypes. Moreover, CMS2 and CMS3 CRCs with intermediate prognosis are mainly distributed in the IS2 type, which also had intermediate prognosis. Nevertheless, it should be pointed out that the CMS cannot clearly predict the prognosis of COAD. In addition, CMS shows a relatively indistinct characterization on the tumor immune microenvironment of COAD. For CMS2–4 CRCs with relatively lower immune infiltration than CMS1, COAD ISs help to show distinct prognosis and more detailed immune characteristics. When compared with the previously defined pan-cancer immune-subtypes based on the data compiled by *TCGA* (24), IS1 and IS2 were most similar to the C1 (wound healing) subtype, which had a poorer prognosis than the other five subtypes in TCGA-COAD dataset. The C6 subtype, with an immunologically suppressed feature and poorest prognosis in all six subtypes in TCGA-COAD dataset, displays the highest TGF-β signature and a high CD4^+^ T-cell infiltrate and is mainly distributed in IS1. The C2 subtypes enriched in many immune-evading related genes and with a high CD8^+^ T-cell infiltrate was mainly distributed in IS3. These results indicate that the three COAD ISs were mapping to different TCGA pan-cancer categories with a similar immune microenvironment. The comparison analysis with other well-established clustering methods demonstrated the reliability of the proposed IS classification. In addition, our data suggest that different and higher-resolution ISs may be useful for better identifying potential recipients of targeted immunotherapies. Our results, therefore, may provide a useful and additional complement in the classification of TME.

Multiple immune checkpoint inhibitors (ICIs) were approved by the FDA for the treatment of patients with unresectable MSI-H or dMMR COAD. From the perspective of TME, MSI-H CRC is mainly of immune-inflammatory type, while MSI-L CRC is insensitive and unlikely to benefit from immunotherapy, and MSS CRC mostly belongs to the immune-privileged type and immune-desert type (56). Several studies have shown that the expressions of cytotoxic cells, CD8+, Th1, Th2, follicular helper T cells, and T-cell markers in MSI-H CRC were significantly higher than those in MSS patients (20). Our results suggest, however, that patients presenting with COAD in different ISs would benefit from IS-specific treatment strategies using ICIs. In TCGA-COAD dataset, most of the MSS patients fell into IS1, while IS3 had the highest percentage of patients with MSI-H. Consistently, by identifying the MMR status and determining the expressions of IFN-γ, GZMB, and CD31 in 223 samples in our FUSCC cohort, we confirmed that patients with a dMMR status had lower IFN-γ and GZMB expressions than patients with a pMMR status. Our IHC results to some extent verified the molecular characteristics of IS1 and IS3.

Moreover, IS1 had the highest TIDE score and the highest predicted T-cell dysfunction score, suggesting that although IS1 is an immune “hot” phenotype, patients may be less likely to benefit from ICIs due to T-cell dysfunction and tumor immune escape. IS1 was predicted to have the highest angiogenesis level. Consistently, our IHC results also showed that the CD31 immunostaining intensity in pMMR cases was higher than that in dMMR cases. Thus, a combination of anti-angiogenic therapeutic drugs with ICIs might have a synergetic antitumor effect for the IS1 type. Being inspired by the “REGONIVO/EPOC1603” trial (57), a phase Ib trial of anti-angiogenetic inhibitors (regorafenib) plus ICIs (nivolumab) for gastric and colorectal cancer, several clinical trials have been established to assess the therapeutic efficacy of a combination of VEGFR/VEGF inhibitors and ICIs in solid tumors, which we believe could benefit patients with IS1 COAD (58). For IS2, the absence of immune cell infiltration consequently represents a non-inflamed TME and so therapeutic strategies that induce immune infiltration may be useful to reinvigorate the immune system in these patients, such as demethylating agents (59), chemo/radiotherapy-inducing immunogenic cell death (60), and tumor vaccines (61). It has been demonstrated that the application of the seasonal influenza vaccine into a tumor facilitates the shift toward a “hot” tumor (16). There is therefore a possibility that this method could be included in the treatment of colorectal cancer, which is highly instructive for the algorithms of treatment for patients with IS2 “cold” tumors. Regarding the 12 prognostic hub genes, given that they belong to the brown module that was negatively correlated with IS2, these genes are the potential targets of a colorectal cancer mRNA vaccine and could be beneficial for patients with IS2. The rich immune cell infiltration of IS3 represents an extremely inflamed TME, making this colorectal cancer subtype most suitable for ICIs (7).

The 12 prognostic hub genes are the major immune genes related to the disease progression risk of IS1 and IS2-COAD, which may serve as potential prognostic and therapeutic markers. Among them, *PDGFRB*-related multitargeted receptor tyrosine kinase inhibitor regorafenib (BAY 73-4506) has been FDA approved for the treatment of metastatic COAD that has progressed after all standard therapies (62). PDGFRB^+^ cancer-associated fibroblasts (CAFs) are an important component of stromal cells in the tumor microenvironment. Previous studies have found that in breast cancer, PDGFRB+CAFs recruit CD4^+^CD25^+^FOXP3^+^ Treg cells, and the recruited Treg cells inhibit the activation and proliferation of CD8^+^T cells in the TME, thereby inducing local immunosuppression (63). In addition, as a pivotal functional molecule in PDGF-BB-PDGFRB signaling, PFGFRB is implicated in the promotion of pericyte–fibroblast transition, which is a propellant for tumor growth and metastasis (64). Similarly, COL5A1, COL6A1, COL6A2, ITGA5, TNS1, and THY1 are markers of CAF activation (65–69). As we present, IS1 has the most abundant stromal content; therefore, stromal cells may play a very important role in this immune “hot” but immunosuppressive phenotype. *CLEC14A* was also a novel anti-angiogenic target for VEGF-dependent angiogenesis and tumor angiogenesis (70). *CLEC14A* is highly expressed in IS1 subtypes, which is consistent with our angiogenesis-related analysis results (IS1 has the highest angiogenesis score) and IHC staining results (CD31 has a higher expression in MSS subtypes of COAD). Blood vessel endothelial cells have long been known to modulate inflammation by regulating immune cell trafficking, migration, and activation (71), and the IS1 subtype is rich in immune cell infiltration. The possible reason is that specific subtypes of endothelial cells participate in immune cell recruitment or direct interaction with immune cells in tissue-specific immunity, which was collectively refer to as “immunomodulatory ECs” (71). Both *AEBP1* (72) and *EFEMP2* (73) have been functionally implicated in malignant tumor behavior and were potential gene therapy targets. The expression level of EFEMP2 is correlated with M0 macrophages infiltrating the TME (74), and our data yielded the same results (the proportion of macrophage M0 and macrophage M2 was significantly higher in IS1 than that in IS2). Considering that IS1 is an immune “hot” but immunosuppressive phenotype, the infiltrated macrophage M0 may be more inclined to polarize to M2 status (switches into the anti-inflammatory M2 phenotype) under the stimulation of various cytokines (such as IL4 and IL-10).

Although further clinical evaluation is required, the potential of these tumor antigens to be successful targets for COADs has been consolidated in these previous reports.

This study provides the conceptual framework of IS for a better understanding of the tumor-specific immune microenvironment of COAD. Stratification of the patients according to the IS system can be used for identifying patients that may respond well to targeted therapies and for designing adequate therapeutic strategies to improve the efficacy of immunotherapy.

## Conclusion

We identified three ISs of COAD that represent distinct clinicopathological, cellular, and molecular characteristics and constructed a robust stable classification method for determining the IS. Immune subtyping could be used to identify COAD patients sensitive to immunotherapy and might guide a personalized approach to cancer immunotherapy.

## Data availability statement

Publicly available datasets were analyzed in this study. This data can be found here: We applied 4 COAD datasets from two online databases: The Cancer Genome Atlas (TCGA, https://portal.gdc.cancer.gov/) database, and Gene Expression Omnibus (GEO) database (including three datasets: GSE39582, GSE17538 and GSE72970; http://www.ncbi.nlm.nih.gov/geo/). We had uploaded all the working sheets as additional files in the submission system.

## Ethics statement

The studies involving human participants were reviewed and approved by Fudan University Shanghai Cancer Center. The patients/participants provided their written informed consent to participate in this study.

## Author contributions

MX, JC, and WnW conceived the study, performed the literature search and bioinformatics analysis, and prepared the figures. XiW, XuW, WiW, CT, MZ, SN, ZD, and LW helped with data collection, analysis, and interpretation. MX, ZH, JC, WW, ZD, and DH wrote and revised the manuscript. MX, JC, and WnW contributed equally to this work. WL, ZD, DH, and WS share the senior authorship of this study. The authors read and approved the final manuscript.

## Funding

This work was supported by the National Natural Science Foundation of China (81972249, 82172702, 81902430), Shanghai Clinical Science and Technology Innovation Project of Municipal Hospital (SHDC12020102), Clinical Research Project of Shanghai Shenkang Hospital Development Center (SHDC2020CR4068), Natural Science Foundation of Shanghai (21ZR1414900, 22ZR1413000), Artificial Intelligence Medical Hospital Cooperation Project of Xuhui District (2021-017), Shanghai Science and Technology Development Fund (19MC1911000), Shanghai Municipal Key Clinical Specialty (shslczdzk01301), Clinical Research Project of Shanghai Municipal Health Committee (20194Y0348), Shanghai “Rising Stars of Medical Talents” Youth Development Program Youth Medical Talents – Specialist Program (SHWSRS(2020)_087), and the Interdisciplinary Program of Shanghai Jiao Tong University (YG2019QNA40).

## Acknowledgements

We would like to thank *TCGA* (http://cancergenome.nih.gov) and the GEO database (https://www.ncbi.nlm.nih.gov/geo/) for data collection. We would like to thank Gao Qin and Wang Zhaoxi from Xinhua Harvard International Healthcare Innovation Collaboration Initiatives for manuscript discussion and review.

## Conflict of interest

The authors declare that the research was conducted in the absence of any commercial or financial relationships that could be construed as a potential conflict of interest.

## Publisher’s note

All claims expressed in this article are solely those of the authors and do not necessarily represent those of their affiliated organizations, or those of the publisher, the editors and the reviewers. Any product that may be evaluated in this article, or claim that may be made by its manufacturer, is not guaranteed or endorsed by the publisher.

## Glossary

**Table:**

APC	adenomatous polyposis coli
APCs:	antigen presenting cells
cGAS	cyclic GMP-AMP synthase
CMS	consensus molecular subtype
COAD	colon adenocarcinoma
ISs	immune subtypes
CYT	immune cytolytic activity
DAVID	Database for Annotation Visualization and Integrated Discovery
DFS	disease-free survival
ESTIMATE	Estimation of STromal and Immune cells in MAlignant Tumours using Expression data
FFPE	formalin fixed paraffin-embedded
FUSCC	Fudan University Shanghai Cancer Center
GRCh38	Genome Reference Consortium Human Build 38
IFN	type I interferon
MAD	median absolute deviation
MCFAUC	comprehensive forecast area under the curve
MSS	microsatellite stable
MSI-L	low microsatellite instability
IARC	International Agency for Research on Cancer
ICIs	immune checkpoint inhibitors
IGP	in-group proportion
IHC	immunohistochemistry
IRF3	IFN regulator 3
pMMR	proficient mismatched repair
PD-L1	programmed death-ligand 1
ROC	receiver operating characteristic
SubMap	subclass mapping
STING	stimulator of interferon genes
TBK1	tank-bound kinase 1
*TCGA*	The Cancer Genome Atlas
GEO	Gene Expression Omnibus
TMA	tissue microarray
TMB	tumor mutation burden
TME	tumor microenvironment
TIDE	tumor immune dysfunction and exclusion
TOM	topology overlap matrix
TPM	Transcripts Per Kilobase of exon model per Million mapped reads
PAM	partition around medoids
WGCNA	weighted gene correlation network analysis
PR	partial response
CR	complete response
HCK	HCK proto-oncogene Src family tyrosine kinase
LCK	LCK Proto-Oncogene Src Family Tyrosine Kinase
	Lymphocyte Cell Specific Protein-Tyrosine Kinase
