# Supplementary material for: Classification of colon adenocarcinoma based on immunological characterizations: Implications for prognosis and immunotherapy
Source: Front Immunol. 2022 Jul 27;13:934083. doi: 10.3389/fimmu.2022.934083 (PMC9363576; doi:10.3389/fimmu.2022.934083)
Supplement: Supplementary file 1 [file DataSheet_1.docx]

# Supplementary Fig. S1 Distribution of IS1-IS3 across clinicopathological characteristics

Distribution of IS1-IS3 across clinicopathological characteristics in TCGA COAD (A), GSE39582 (B) and GSE17538 (C) cohorts.

# Supplementary Fig. S2 Association between immune subtypes and TMB and gene mutation

(**A, B**). TMB (A) and mutation (B) number in COAD IS1-IS3 in *TCGA*-COAD cohort.

(**C**). Top ten highly mutated genes in ISs.

# Supplementary Fig. S3 Identification of immune gene co-expression modules of COAD.

(**A**). Sample clustering. (**B**). Scale-free fit index for various soft-thresholding powers (β). (**C**). Mean connectivity for various soft-thresholding powers.

**Supplementary Table S1**. The enrichment score of immune characteristics in TCGA-COAD dataset.

**Supplementary Table S2**. The enrichment score of immune characteristics in GSE39582 dataset.

**Supplementary Table S3**. The enrichment score of immune characteristics in GSE17538 dataset.

**Supplementary Table S4**. The prognostic significance of the enrichment score of each immune characteristic in TCGA-COAD dataset

**Supplementary Table S5**. The prognostic significance of the enrichment score of each immune characteristic in GSE39582 dataset

**Supplementary Table S6**. The prognostic significance of the enrichment score of each immune characteristic in GSE17538 dataset

**Supplementary Table S7**. Supplementary Table S7. Genes' mutation frequency in each ISs

**Supplementary Table S8**. Genes with significantly high mutation frequency in at least one of all three ISs

**Supplementary Table S9**. Genes list in the 22 co-expression modules

**Supplementary Table S10**. Cox survival regression analysis of genes in the brown module

**Supplementary Table S11**. Cox survival regression analysis of genes in the darkolivegreen module
