# Supplementary figures and images for: Classification of colon adenocarcinoma based on immunological characterizations: Implications for prognosis and immunotherapy
Source: Front Immunol. 2022 Jul 27;13:934083. doi: 10.3389/fimmu.2022.934083 (PMC9363576; doi:10.3389/fimmu.2022.934083)

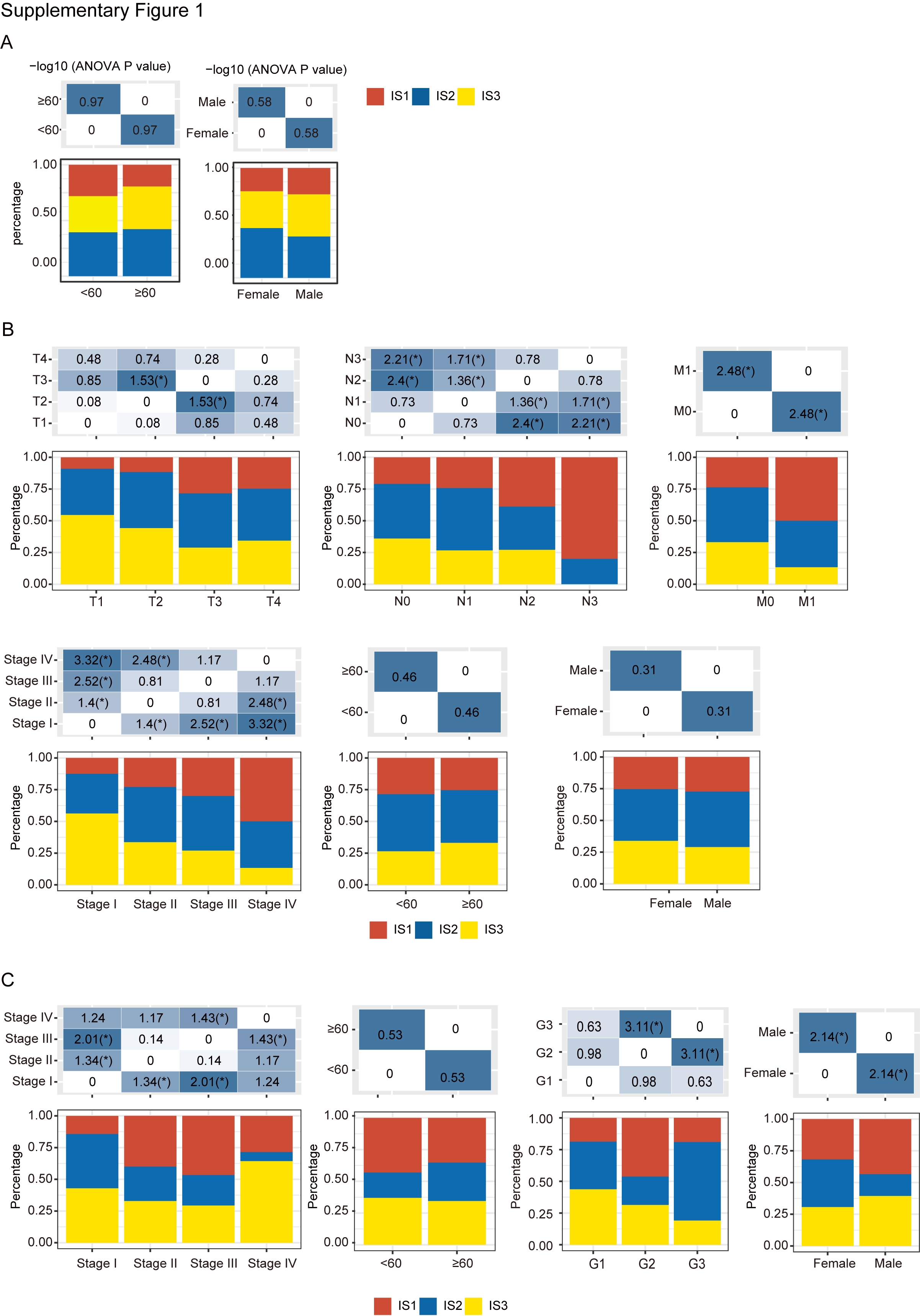

Supplement: Supplementary file 2 [file Image_1.tif]

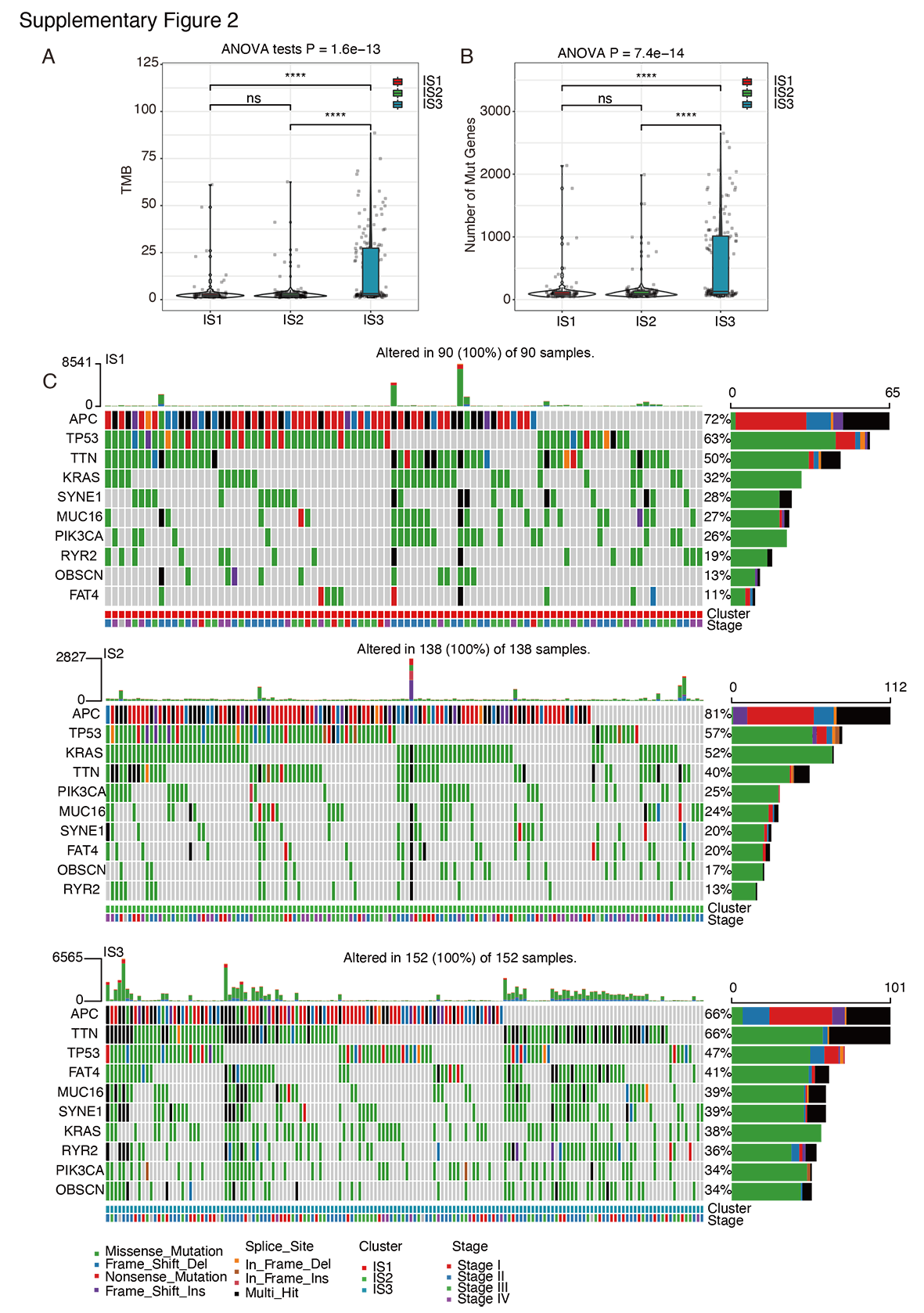

Supplement: Supplementary file 3 [file Image_2.tif]

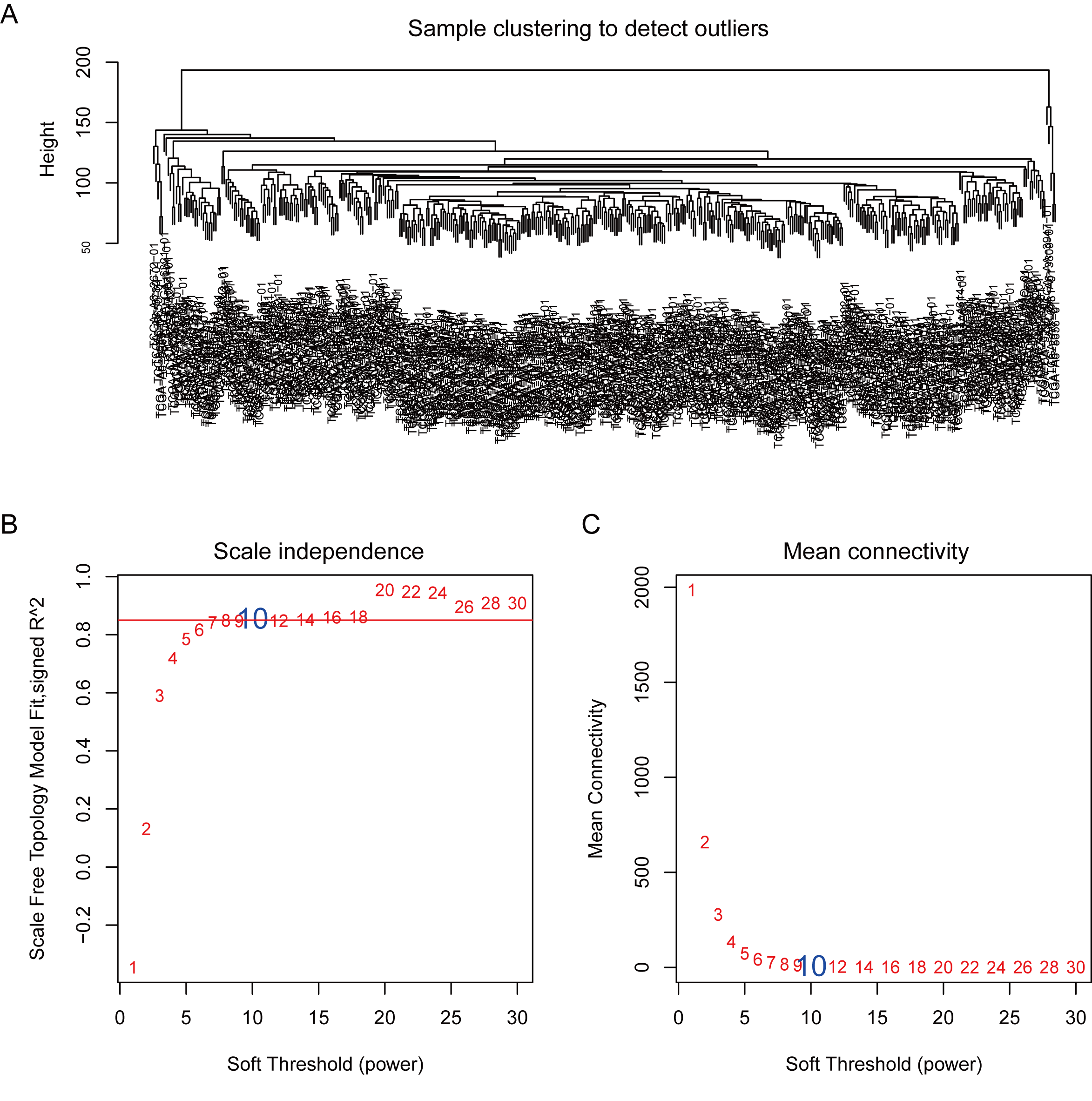

Supplement: Supplementary file 4 [file Image_3.tif]
